# Evolutionary Approach of Intrinsically Disordered CIP/KIP Proteins

**DOI:** 10.1038/s41598-018-37917-5

**Published:** 2019-02-07

**Authors:** Muhamad Fahmi, Masahiro Ito

**Affiliations:** 1 Advanced Life Sciences Program, Graduate School of Life Sciences, Ritsumeikan University, 1-1-1 Nojihigashi, Kusatsu, Shiga, 525-8577 Japan; 2 Department of Bioinformatics, College of Life Sciences, Ritsumeikan University, 1-1-1 Nojihigashi, Kusatsu, Shiga, 525-8577 Japan

## Abstract

The mammalian CIP/KIP family proteins are intrinsically disordered proteins (IDPs) that can regulate various cellular processes. However, many reports have shown that IDPs generally evolve more rapidly than ordered proteins. Here, to elucidate the functional adaptability of CIP/KIP proteins in vertebrate, we analysed the rates of evolution in relation to their structural and sequence properties and predicted the post-translational modification based on the sequence data. The results showed that CIP/KIP proteins generally could maintain their function through evolution in the vertebrate. Basically, the disordered region that acts as a flexible linker or spacer has a conserved propensity for structural disorder and a persistent, fast rate of amino acid substitution, which could result in a significantly faster rate of evolution compared to the ordered proteins. Describing the pattern of structural order-disorder evolution, this study may give an insight into the well-known characteristics of IDPs in the evolution of CIP/KIP proteins.

## Introduction

The CIP/KIP family is one of two cyclin-dependent kinases (CDKs) inhibitor (CDI) protein families and it includes p21^Cip1/Waf1/Sdi1^, p27^Kip1^, and p57^Kip2^(refs^1–6^). The family members can be distinguished into another CDI protein family by a conserved N-terminal domain called a CDI domain or kinase inhibitory domain (KID), which allows them to regulate cell cycle progression by binding to both cyclin and CDK subunits of mitogens, whereas INK4 members bind to cyclin only^1,7^. Although CIP/KIP members have similar specificities on their CDI/KID, they have shown to have distinct functions and regulatory mechanisms related to the divergence in their remaining sequence^1,7^. Correspondingly, CIP/KIP members have a different motif near their C-terminal, in which p21, p27, and p57 have proliferating cell nuclear antigen (PCNA)-binding motif, a stretch of conserved glutamine and threonine residues namely QT-box motif, and both PCNA-binding motif and QT-box motif, respectively^6,8,9^. Initially recognised as inhibitors of cell cycle progression, CIP/KIP proteins have also been reported to be involved in many other regulatory mechanisms, including transcriptional regulation^7^, cell migration^10^, and apoptosis^11^. Furthermore, recent studies have indicated that all CIP/KIP proteins also act as tumour suppressors and may be oncogenic under certain conditions^1^. The multifaceted abilities of these proteins may originate from their partial or complete lack of a cooperative, folded structure under native conditions, namely intrinsically disordered proteins (IDPs)^12–18^. However, it was reported that IDPs generally evolve more rapidly than ordered proteins^19,20^. Therefore, to understand how these proteins get along with the IDP fast rates of evolution, it is critical to examine their evolution in relation to structural features.

Generally, the primary sequence of IDPs is composed of a high proportion of charged and polar amino acids and is deficient of bulky hydrophobic amino acids^21,22^. These characteristics may result in a flexible conformation of disordered regions, which are unable to fold spontaneously into three-dimensional structures and tend to adopt specific tertiary conformations, at least locally, only after binding to their partners^21–23^. The molecular interactions of IDPs can be transient and dynamic, such as to function as switches fine-tuned by post-translational modifications (PTMs) and to function as chaperones. On the other hand, it also can be permanent, such as those that enable them to modify the activity of other proteins, assemble multiple binding partners to promote protein complexes, and function as scavengers, which store and neutralize small ligands^21,24–26^. In addition, disordered regions can also retain their structure without ever becoming structured or showing the so-called “fuzziness” phenomenon and may function as flexible linkers or spacers^23,26,27^. Accordingly, these characteristics have consequences for IDP ability to exhibit functional promiscuity under different conditions^23^.

Because conformationally flexible proteins tend to have functional promiscuity, studies on IDPs evolution have attracted a lot of attention, particularly with regards to the rates of amino acid substitution compared with ordered proteins. Many reports have shown that IDPs generally evolve more rapidly than ordered proteins^19,20^. These differences may be due to differences in amino acid compositions, intramolecular contacts, and functional differences^19,23,28^. Additionally, recent assessments have shown that disordered proteins have different accepted point mutations and higher insertion and deletion rates compared with ordered proteins^29^. Disordered regions are also associated with more modification sites than ordered regions, particularly phosphorylation^30^. Given the ability to mediate multiple biological processes, CIP/KIP proteins also have a higher tendency to evolve over time. Thus, the evolutionary mechanism of CIP/KIP proteins may provide important information about genome evolution, in general. Here, to elucidate the functional adaptability of CIP/KIP proteins, we calculated the rates of evolution per site and the rates of structural properties transitions, and predicted their structural disorder and secondary structure propensities. In addition, we also predicted and examined the phosphorylation and the conserved phosphorylation sites, respectively.

## Results

### Evolution of structural order-disorder and secondary structure

**Figure 1:**
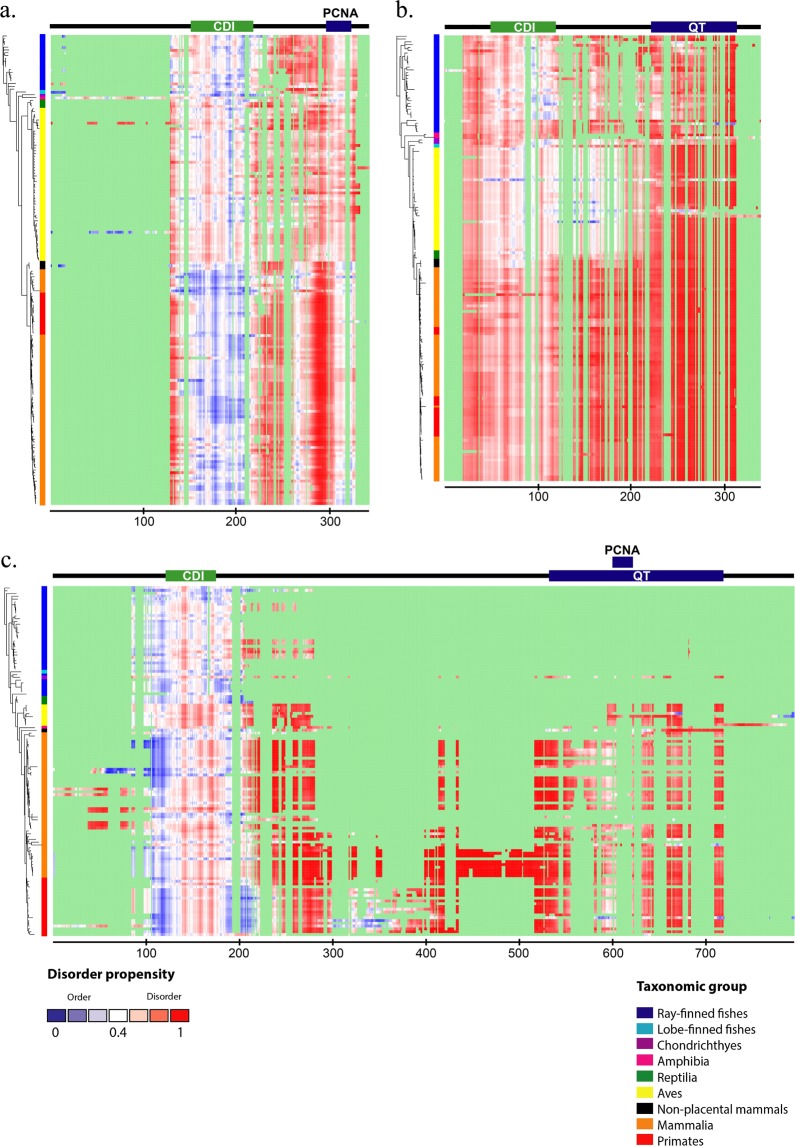
The order-disorder propensity of each CIP/KIP protein across vertebrates. The heat maps of order-disorder propensity based on the IUPred prediction score are plotted following the position of multiple sequence alignments (columns) and the taxa position in the phylogenetic tree (rows). The heat maps show a colour gradient from blue (ordered) to red (disordered) with white as the boundary between the two propensities, and a light green as gaps. Coloured boxes between the trees and heat maps indicate the taxonomic group. There is a domain and motif bar above the heat map of each protein. In the domain and motif bar, the green area indicates the CDI/KID domain, and blue area indicates either PCNA-binding motif or QT-box motif, whereas the black line indicates no important region. Three heat maps, p21 **(a)**, p27 **(b)**, and p57 **(c)**, and a colour guide for the taxonomic group are shown in the figure.

**Figure 2:**
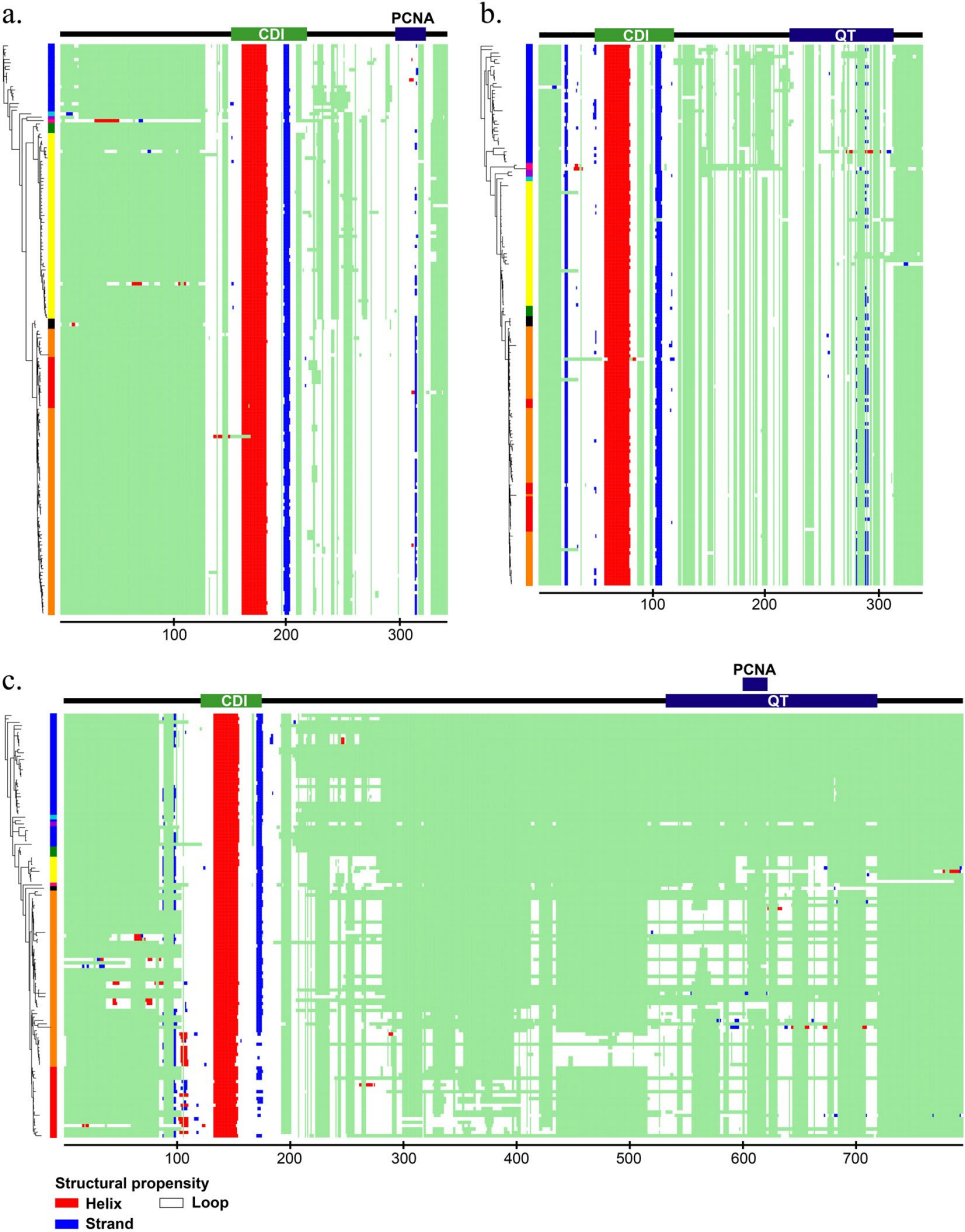
The secondary structure propensity of each CIP/KIP protein across vertebrates. The heat maps of secondary structure propensity based on the Jpred4 prediction score are plotted following the position of multiple sequence alignments (columns) and the taxa position in the phylogenetic tree (rows). The heat maps show a fixed of red (helix), blue (strand), and white (coil), and a light green as gaps. Coloured boxes between the trees and heat maps indicate the taxonomic group following the taxonomic group reference (Fig. 1). There is a domain and motif bar above the heat map of each protein. In the domain and motif bar, the green area indicates the CDI/KID domain, and blue area indicates either PCNA-binding motif or QT-box motif, whereas the black line indicates no important region. Three heat maps, p21 **(a)**, p27 **(b)**, and p57 **(c)**, are shown in the figure.

**Figure 3:**
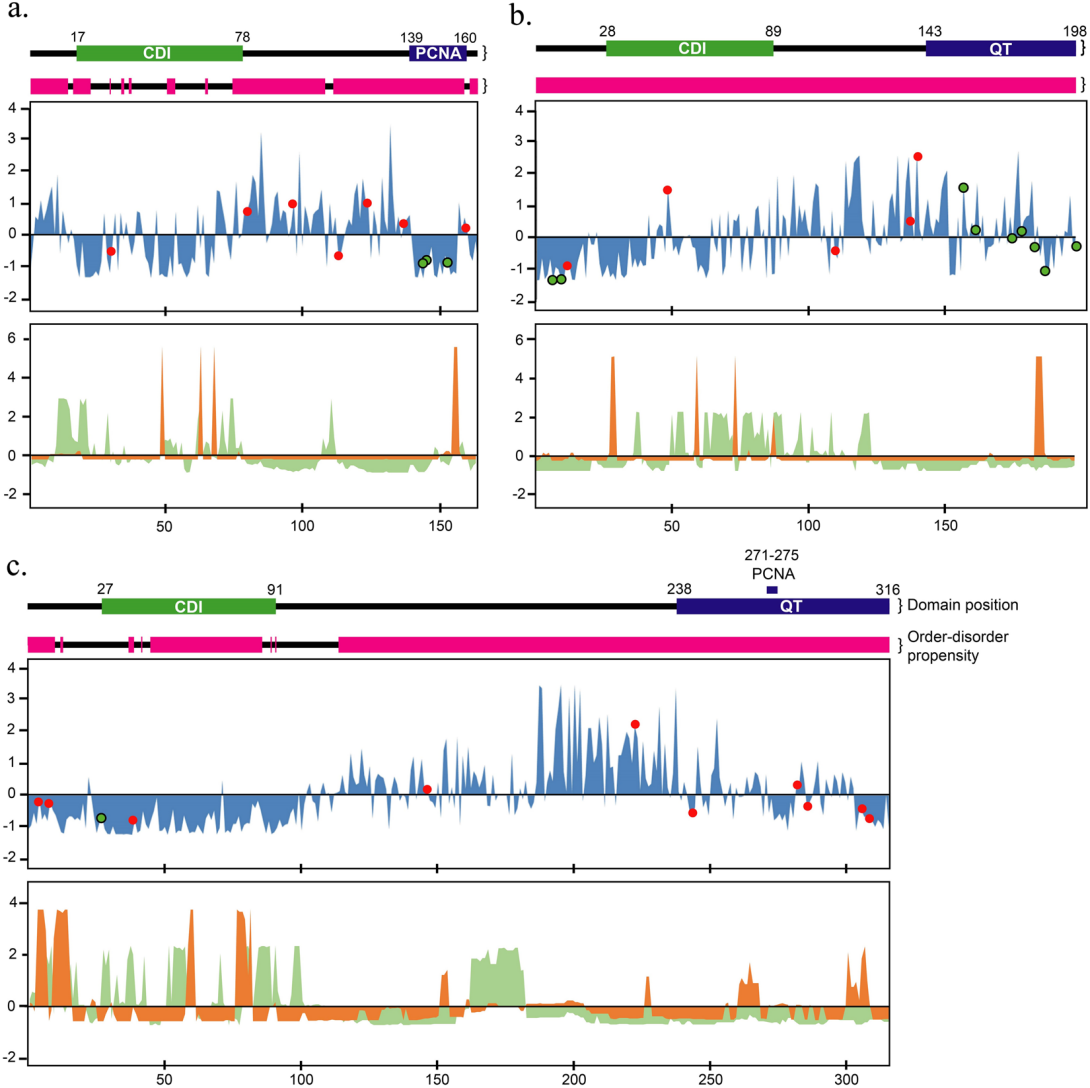
Rates of evolution per site with predicted phosphorylation sites of human CIP/KIP proteins and evolutionary dynamics of predicted secondary structures and structural order-disorder. There are two graphics on each sub-figure, the rate of evolution per site on the above and the evolutionary dynamics of predicted structural properties on the below. In the rate of evolution graphic, the rates of amino acid substitution are shown as blue area chart. The black-bordered green dots indicate the conserved phosphosites, following the 50% majority rule of the predicted site, whereas the red dots indicate non-conserved phosphosites. There are two bars above each graphic, the domain and motif bar and order-disorder propensity bar. In the domain bar, the green area indicates the CDI domain, and blue area indicates either PCNA or QT-box motif, whereas the black line indicates no important region. In the order-disorder propensity bar, the magenta area indicates disordered structure, and black line indicates ordered structure. In the evolutionary dynamics of structural properties, the rates of secondary structure transition and order-disorder transition are shown as orange and green area chart, respectively. There are three graphics, p21 (**a**), p27 (**b**), and p57 (**c**), shown in the figure with x-axis as the sequence length and y-axis as the Z-score of the rate of evolution.

To investigate the evolutionary patterns of structural properties of the CIP/KIP members in vertebrate, we predicted the propensity of structural order-disorder and secondary structures and mapped the results of which to the taxon position in the phylogenetic tree and multiple sequence alignment (Figs 1 and 2). We also calculated the transition rates of the structural order-disorder and secondary structure propensity to investigate the dynamic evolution of those properties (Fig. 3). The results might comprehensively show the dynamics changes of proteins structures which is highly related to function. In general, each protein had a specific evolutionary pattern of the structural properties, and p57 showed to have an apparent distinct pattern to the other members.

The CDI/KID domain was encountered through pfam for all CIP/KIP members in all species, and there were two CDI/KID domains found for p21 of amphibian. This domain of CIP/KIP members which is responsible to control cell-cycle progression in mammals generally had a similar and conserved secondary structure propensity, with a little distinction in the transition patterns and rates (Fig. 2). In this domain, only the p57 of strand secondary structure showed to have obvious repeated transitions from mammals to primates (Fig. 2c). On the other hand, each CIP/KIP member showed to have a specific order-disorder propensity pattern through evolution in this domain. The order-disorder propensity of p21 CDI/KID domain was relatively not conserved and was characterised by a major transition to disorder from reptiles and birds to mammals, non-placental mammals, and primates; the remaining taxa had nearly random patterns (Fig. 1a), whereas p27 (Fig. 1b) showed to have a conserved propensity of structural disorder with a few order propensities observed without a significant pattern, and p57 (Fig. 1c) was commonly composed of a disordered propensity with frequent transitions to order. Despite the CDI/KID domain, the CIP/KIP members also have an important region in their C-terminal fragments. Here, p21 and p57 have a proliferating nuclear antigen (PCNA)-binding motif, and p27 and p57 share, a structural motif that is likely to function in protein-protein interaction, a QT-box motif. The secondary structure prediction results showed that the PCNA-binding motif of p21 and QT-box motif of p27 have a strand secondary structure with many transitions (Fig. 2a,b), whereas no major pattern of secondary structure have found for either PCNA-binding motif or QT-box motif of p57 (Fig. 2c). Further, the order-disorder propensity prediction showed that the functional C-terminal fragments of all CIP/KIP members are conserved to be disordered (Fig. 1), and the region between CDI/KID domain and C-terminal fragment of all CIP/KIP members showed to have no prominent secondary structure property and conserved to be disordered (Figs 1 and 2). The faster rates of structural properties transitions were commonly distributed in the CDI/KID domain and C-terminal fragments (Fig. 3).

Based on the distribution of the structure propensity score in all regions, p27 tended to be more disordered than p21 and p57, with scores of 0.523 ± 0.179, 0.685 ± 0.177, and 0.601 ± 0.241 (means and standard deviations) for p21, p27, and p57, respectively (Supplementary Fig. S1b). In addition, for the CDI/KID domain, the means and standard deviations were 0.386 ± 0.101, 0.535 ± 0.106, and 0.474 ± 0.113 for p21, p27, and p57, respectively (Supplementary Fig. S1a). Thus, on an average, the frequency of the sequence that tends to be ordered is greater in the CDI domain than in the entire sequence for all CIP/KIP members. Each human CIP/KIP protein exhibits a different genetic distance pattern relative to the vertebrate class. As shown in the p-distance value analysis, compared with the human sequence, chondrichthyes had the most diverse sequence identity for p21 protein (59.8%), whereas p27 was the most diverse in amphibia (54.3%) and p57 was the most diverse in ray-finned fish (51.4%). p27 had the lowest genetic distance value for each class, except amphibia (Supplementary Tables S1, S2, and S3). Thus, p27 had the slowest rate of evolution of all CIP/KIP proteins.

### Evolution rates of sequences and modification sites

To investigate the relationship between rates of amino acid substitution and the structural properties and function, we calculated the rates of amino acid substitution. The results are Z-scores, which mean the scores of more than zero is evolving faster than average, and the scores of less than zero is evolving slower than average. In addition, we also predicted the sites of post-translational modification (PTM), i.e., phosphorylation, in the vertebrate and examined the modification site conservation through species evolution following the 50% majority rule. This approach was used to investigate the contributions of protein modifications to the rates of amino acid substitution.

In general, each protein had its own specific distribution of the rates of evolution. However, similar patterns were observed in relation to the functional region. Generally, but not always, conserved modification sites and the adjacent region have a lower rate of amino acid substitution (Fig. 3). However, there are still exceptions to such phenomena at a few conserved modification sites in p27. Three conserved modification sites were found to have a faster rate of amino acid substitution, with one modification site having a markedly faster rate. In addition, for modification sites that are not considered conserved, many sites located in the disordered region had a lower rate of substitution than average. However, sites having faster substitution rates were also abundant. In this case, conserved modifications were more reliable for slowing amino acid substitution rates in disordered regions, which generally had a faster rate of evolution. On the other hand, excluding sites with conserved modifications and their surrounding regions, CDI/KID domain of all CIP/KIP proteins have, generally, a lower rate of evolution than average. Further, the PCNA-binding motifs of p21 and p57 also have similar results of low evolution rates. On the other hand, the QT-box motif of p27 and p57 showed both lower and faster rates of amino acid substitution than average, whereas their structures were constrained to be disordered, and the linker region of CDI/KID domain and the C-terminal motifs commonly have faster rate of evolution with conserved disorder propensity.

Regarding the modification site prediction results, 14 phosphorylation sites had been predicted to occur in the human p27 sequence, whereas 10 and 11 sites had been found for p21 and p57, respectively. Most modification sites were found in the disordered region outside of the CDI/KID domain (Supplementary Fig. S2). Only two sites located in the CDI/KID domain were found, one from p21 and another from p57. In contrast, p27 possessed a higher number of predicted phosphorylation sites than other protein members, and it also possessed the highest number of conserved phosphorylation sites as well. In human p21, there were T145, S146, and S153 that showed to be conserved in propensity and sequence to a vertebrate; on the other hand, there were S7, S10, S12, T42, S110, S138, S140, T157, S161, S175, S178, S183, T187, and T198 in human p27; while there were only S28 in human p57. Except for S7, S161, S175, S178, and S183 in p27 and S28 in p57 (Fig. 3), the conserved phosphosites based on the NetPhos predictions were shown to have a well-known function based on the experimental data in the PhosphoSite+ database (as at Aug. 2018).

## Discussion

The structural studies of p21 using proteolytic mapping, circular dichroism (CD) spectropolarimetry, and nuclear magnetic resonance (NMR) revealed that the protein and CDI/KID domain are lack of folded structure^16^. In addition, the structural studies of p57 and p27 also showed the same characteristic of the highly disordered structure as shown by CD and fluorescence spectra, and NMR and molecular dynamics (MD) analysis, respectively^12,14,17^. Consistent with this, the prediction of order-disorder propensity of CIP/KIP proteins in this study also showed that these proteins are highly disordered in structure. Further, their rates of amino acid substitution showed a relatively similar distribution, and some disordered regions involved in intermolecular interactions and post-translational modifications were constrained. Describing the pattern of structural order-disorder evolution, this study may give an insight into the well-known characteristics of IDPs in the evolution of CIP/KIP proteins.

**Figure 4:**
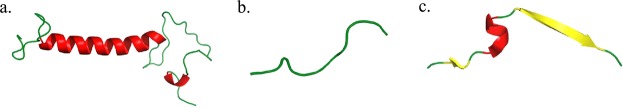
Structures of some important fragments in CIP/KIP proteins. Structure of p27 CDI/KID from MD analysis^17^ **(a)**, p27 C-terminal fragments (aa 181–190)/S-phase kinase-associated protein 1 A (SKP1)/S-phase kinase-associated protein 2 (SKP2)/Cyclin-dependent kinases regulatory subunit 1 (CKS1) complex from X-ray diffraction^63^ **(b)**, and p21 C-terminal fragments (aa 141–160)/PCNA complex from X-ray diffraction^64^.

Interesting results were shown in the CDI/KID domain, in which all CIP/KIP proteins noted to have a conserved secondary structure in all vertebrate sample, and both p21 and p57 have several transitions of order-disorder propensities in the evolution of vertebrate. The structural studies of these proteins revealed that the CDI/KID domain is likely to have a transiently populated secondary structure which may exhibit a pre-molten globule-like of structure (Fig. 4a)^16,17^. Even though this region, particularly in p21 and p57, were often predicted to be ordered in this study, the structural studies reported that this domain is lack of folded structure. The characteristics of these fragments that could pre-formed structural elements are likely to result in the order propensity in the prediction method. On the other hand, even though the order-disorder transitions were commonly centered in this region based on the prediction, the CDI/KID domain of all Cip/Kip proteins generally had lower rates of evolution than average, despite there were still some other sites that showed faster rates than average. The CDI/KID domain itself is the binding site for regulating cyclin and CDK complexes^1^; thus its structure could be altered through the balance between the specificity and affinity of cyclins and CDKs throughout evolution. Evolutionarily, cyclins show great diversification and complexity in various organisms ranging from protists to animals. Likewise, the numbers of CDKs have also increased throughout species evolution as well as cyclins^31^. The faster rate of amino acid substitution found in the CDI/KID domain is likely to be an important site for adapting the affinity to the binding partner. However, the generally conserved sequences found here suggested that the CDI/KID domain still maintain their specific structures in the vertebrate.

Another important region of the CIP/KIP proteins is located near the C-terminal, of which p21 has proliferating nuclear antigen (PCNA)-binding motif (Fig. 4c) and p27 and p57 share a QT-box motif with a low similarity (Fig. 4b)^8,32,33^. In addition, p57 was also found to interact with PCNA (via aa 271–275), with a lower affinity than p21 (ref.^33^). PCNA itself appears to be conserved throughout evolution, and most plant PCNAs exhibit features similar to those found in PCNAs of other eukaryotes^34^. Thus, the PCNA-binding motif of p21 and p57 might be constrained to maintain the binding affinity. The short linear motif (SLiM) displays a common functional module in the disordered region. It can bind to the globular domains helped by flexible structure around them^26^. Accordingly, both proteins have a similar critical function in their interaction with PCNA; both proteins have an ability to inhibit PCNA activity and ability in DNA replication, thereby facilitating the role of the cell repair machinery throughout the cell cycle^7,33^. Further, the PCNA-binding motifs of both p21 and p57 have the constrained disordered structures in vertebrates, even though the structural secondary structure prediction showed that p21 PCNA-binding motif commonly have a strand propensity. These supported the well-known characteristics of IDP that essential intermolecular interactions and conserved binding partners could constrain disordered regions through evolution.

The QT-box motif showed to have both lower and faster rate of amino acid substitution than average in both p27 and p57, even though its structure was constrained to be disordered. The heterogeneity of the rates of amino acid substitution here likely displays that this region contains multiple short linear motifs (SLiMs). The QT-box is a structural motif that previously reported to function in protein-protein interaction^6^. Predicted to be conserved in disordered structure, the fragments that have lower rates of evolution are relevant to mediate the protein-protein interaction. For p27, many conserved and functional phosphorylation sites, which determine various fates including cytoplasmic localization, stability, and degradation, lie in the QT-box motif^35,36^. Likewise, for p57, besides the existence of a PCNA-binding motif inside the QT-box motif, an important phosphorylation site, T310, for protein degradation is also located in this domain^8,37^. Since there are many important phosphosites in the QT-box motif, flexibility may be required in the structure of this domain to keep those sites exposed. In addition, functional phosphosites and binding sites need to be constrained in order to maintain the function.

All human CIP/KIP proteins have important regions near the N-terminal and C-terminal that are separated by regions whose rate of amino acid substitution, for each sequence, is commonly faster than average for all vertebrate species. However, despite their faster rate of substitution, these regions were constrained to have a disordered structure for all vertebrate species, except for the more primitive mammalian species with p57, which does not have these regions. Based on the sequence composition, disordered regions generally have more charged and fewer aromatic amino acids as well as faster rates of amino acid substitution than ordered regions^22,38,39^. Thus, it suggested that the substitution of amino acid in this region of CIP/KIP proteins is constrained to a specific residue. The conservation of disordered structure having rapid amino acid substitution rates may be related to their functions as flexible linkers that allow movement of the important region positioned on either end or spacers which regulate the distances between important region^26^. Indeed, this mechanism of evolution seems to only maintain the flexible properties of the region. Here, the constrained sequences may not be necessary, as the mutation yields a sequence that is flexible in structure. Accordingly, there may still be a selection for faster rates of substitution in disordered regions. This characteristic may be the main reason why disordered proteins generally have faster rates of evolution than ordered proteins.

Interestingly, the CIP/KIP family has two different mechanisms of evolution in maintaining the length of the sequence, particularly in disordered regions from after the CDI/KID domain to the C-terminal. Generally, disordered regions also tend to occupy repeat expansions and to have more insertions and deletions than ordered regions despite the faster rate of amino acid substitution^26,29^. Frequent repeat expansions, insertions, and deletions could create an obvious divergence in the sequence length over evolution. However, only p57 showed a clearly different sequence length over evolution in its disordered region. In contrast, p21 and p27 exhibited high rates of amino acid substitutions without any obvious differences in sequence length. In addition, the PCNA-binding motif and QT-box motif of p57 also could only be detected from aves to mammals and primates based on the multiple sequence alignment. What are the factors that make p57 to occupy similar motifs to other CIP/KIP members in the evolution process of vertebrates? the elucidation of the actual factors responsible for the distinct frequency of insertions and deletions between these proteins clearly needs further studies.

The promiscuity of IDPs is highly affected by post-translational modifications (PTMs), particularly phosphorylation, which influences the binding affinity of disordered proteins^28,40^. In addition, for example in yeast, disordered proteins are associated with twice as many kinases as structured proteins on an average^30^. Phosphorylation plays important roles in protein function and is critical for biological processes and subcellular protein localisation^41^. Therefore, the evolution rates of phosphosites are assumed to be different from those of nonphosphorylated residues in the disordered region. Based on the predictions here, several of the predicted phosphosites of human CIP/KIP proteins were shown to have a lower rate of amino acid substitution. Some of them are well-studied phosphosites whose functions are known and have a conserved propensity to be phosphorylated; however, some phosphosites showed a different pattern; in particular, T157 in p27 that showed to have a higher rate of amino acid substitution. On the other hand, some well-known functional phosphosites of CIP/KIP proteins in humans were shown to have a higher rate of evolution but were not conserved to be phosphorylated in vertebrates. Therefore, in CIP/KIP proteins, the existence of phosphosites in the disordered region may constrain the rate of evolution.

In human p21, T145, S146, and S153 showed a conserved propensity to be phosphorylated in the vertebrate. Each of these phosphosites has an important function in modulating either cytoplasmic relocalisation, protein degradation, or protein stability of p21^42–44^. In human p27, the number of conserved phosphosites was higher than that in the other Cip/Kip proteins; however, some of the conserved phosphosites, including S7, S161, S175, S178, and S183, have not yet been well-studied. Interestingly, the well-studied conserved phosphosites in this protein, S10, T157, T187, and T198, have a similar function to the human p21 conserved phosphosites. The activities of p21 and p27 are controlled by their concentration and subcellular localisation, and modulating these factors may expose the proteins to a different range of potential binding partners. Thus, phosphosites modulating essential functions may be conserved. However, identifying other phosphosites that have overlapping functions must also be a concern, since some of the non-conserved human p21 and p27 phosphosites were also shown to have a similar function to the conserved phosphosites of each protein, even though they are modulated by different signals; for example, T57 and T80 in human p21 that modulate degradation^43,45^.

On the other hand, based on the phosphorylation propensity, the single conserved phosphosite of p57 found here, S28, has not yet been well-studied. Currently, only S282 and T310 of human p57 have been well-studied^37,46^. However, only T310 was shown to have a lower rate of amino acid substitution than the whole site on average. Since p57 has a frequent insertion in vertebrates throughout evolution, the functional modification sites may have been shifted via the unstable length of the linkers, and thus, making it difficult to trace the conserved function through evolution. Therefore, an extensive amount of insertions and deletions in disordered regions could affect the perceived rate of evolution of modification sites, particularly at greater divergence times^29^.

It is important to remember that the properties of structural disorder and phosphorylation sites of CIP/KIP proteins in this study have been inferred using linear sequence predictors. Therefore, the results in this study cannot be interpreted as a perfect or true depiction of the nature of IDPs. However, since experimental data is not available, these results can still be used and considered as the viewpoint of computational biology.

In conclusion, the disordered region in CIP/KIP proteins that exposes many functional phosphosites and binds to various regulators is generally essential for the multifaceted biological roles of CIP/KIP proteins. These regions of CIP/KIP proteins frequently have lower rates of evolution and could conserve the structural properties. The CIP/KIP disordered region that is placed between the important regions in the N-terminal and C-terminal has a conserved disorder propensity with high rates of evolution. The presence of this type of region could result in a significantly faster rate of evolution compared to the ordered proteins. Considering this study was based on prediction, it cannot be given as a true depiction. Therefore, further study related to the dynamic nature of IDPs is required. However, this study could give an insight into the well-known characteristics of IDPs in the evolution of CIP/KIP proteins.

## Methods

### Sequence retrieval, alignment, and pairwise distance

Vertebrate sequences were collected for each CIP/KIP protein, including p21, p27, and p57, from the NCBI database using NCBI BLAST (ref.^47^) under the blastp (protein-protein BLAST) algorithm (Supplementary Dataset 1). For each CIP/KIP protein, the human protein sequence (p21: CAG38770.1; p27: CAG33680.1; p57: P49918.1) was used as the reference to obtain vertebrate sequences in the RefSeq database (ref.^48^). Only one sequence with the highest Max score of BLAST for each species was used for each CIP/KIP protein to minimise redundancy. For alignment, whole vertebrate sequences and representative organism sequences were constructed for each dataset of CIP/KIP proteins. The species included in the representative dataset were *Homo sapiens*, *Rhinopithecus roxellana*, *Mus musculus*, *Sarcophilus harrisii* (for p21 and p27), *Plethodon cinereus* (for p57), *Chrysemys picta bellii*, *Gallus gallus*, *Latimeria chalumnae*, *Danio rerio*, *Xenopus laevis* (for p21 and p27), *Nanorana parkeri* (for p57), *Callorhinchus milii* (for p21 and p27), and *Rhincodon typus* (for p57). All datasets were aligned using MAFFT version 7 (ref.^49^). The iterative refinement method L-INS-I was used for the alignment strategy with maximum of 1000 iterations. Pairwise distance was calculated for representative data alignment of each CIP/KIP protein using Mega7 (ref.^50^).

### Phylogenetic analysis

Phylogenetic trees were constructed using Bayesian inference from each dataset of vertebrate CIP/KIP protein alignment. The trees were estimated using MrBayes 3.2 (ref.^51^) in the CIPRES Science Gateway (ref.^52^), with Jones, Taylor, and Thornton^53^ amino acid substitutions with + F method and gamma shape parameter (JTT + F + G) model which was selected as the best fit model under Akaike Information Criterion (AIC) by Aminosan (ref.^54^) for all vertebrate datasets. The analysis used four simultaneous metropolis-coupled Markov chain Monte Carlo for 5,000,000 generations with a sampling frequency of 1,000 generations. The consensus topology trees were calculated by discarding the first 25% of trees using the 50% majority rule.

### Structural disorder prediction

The structural disorder propensity of all unaligned vertebrate datasets was predicted using IUPred (ref.^55^) with the option for long disordered regions. This prediction has values ranging from 0 (strong propensity for an ordered structure) to 1 (strong propensity for a disordered structure), with 0.5 as the cut-off between order and disorder. However, we used 0.4 as the order-disorder propensity cut-off instead of 0.5 in this study because the former value is considered more accurate in predicting the experimentally verified disordered structure in the DisProt database than the original cut-off ^56^. The order-disorder propensity scores were mapped onto the multiple sequence alignment and taxon position in the tree, and visualised in a heat map using iTOL (ref.^57^). Further, the order and disorder propensity results were also converted into binary matrix, 0 and 1, respectively, to analyse the evolutionary dynamics of structural order-disorder.

### Phosphorylation site prediction

Phosphorylation site was predicted using NetPhos (ref.^58^) for all sequences in the datasets. A site having a value of greater than or equal to 0.75 was predicted to be phosphorylated, whereas a value of less than 0.75 was predicted to be not phosphorylated. The prediction results for each sequence were plotted following the multiple sequence alignment and phylogenetic tree of each dataset. Furthermore, the predicted phosphorylated site of the human sequence was considered to be conserved through evolution if it was predicted to be phosphorylated in the vertebrate sequence following the 50% majority rule of the amount of sequence in the alignment.

### Secondary structure prediction

Secondary structure was predicted using Jpred 4 (ref.^59^). This tool incorporates the Jnet algorithm and has 82.0% accuracy in predicting secondary structure^59^. The prediction was performed for all sequences in each dataset. The results of the prediction were mapped and visualised in a heat map. In addition, the results of secondary structure propensity were also converted into binary matrix, 1 for helix and strand, and 0 for loop, to examine the evolutionary dynamics of secondary structure-loop transitions.

### Evolution rates of sequence data

The substitution rates of amino acids for each vertebrate dataset alignment were analysed using Rate4Site (ref.^60^). The analysis was performed using the empirical Bayesian principle with the Jones, Taylor, and Thornton amino acid substitution model and 16 discrete categories of the prior gamma distribution. The Bayesian inference tree of each dataset was used for the input tree, and the branch lengths were not optimised. Human CIP/KIP protein was used as the reference sequence. Gap was treated as missing data based on sequence reference.

### Evolutionary dynamics of predicted secondary structures and structural disorder

The evolutionary dynamics was investigated by calculating the evolution rates based on the phylogenetic trees and binary matrices for each secondary structure prediction and structural order-disorder prediction. This analysis was used to study the gain/loss transitions of the structural property by adopting the phyletic patterns^61^. Gain Loss Mapping Engine (GLOOME) was used with equal gain and loss and 6 categories of gamma rate distribution^62^. The Bayesian inference tree and human sequence for each dataset were used for the input tree and the reference sequence, respectively. The gap was treated as missing data, and the outputs of evolution rates were standardized as Z-score.

## Supplementary information

Supplementary figure 1, Figure 2, Table 1, Table 2, and Table 3

Dataset 1

## References

[1] Besson A, Dowdy S, Roberts J (2008). CDK inhibitors: cell cycle regulators and beyond. Developmental Cell.

[2] Harper JW, Adami GR, Wei N, Keyomarsi K, Elledge SJ (1993). The p21 Cdk-interacting protein Cip1 is a potent inhibitor of G1 cyclin-dependent kinases. Cell.

[3] El-Deiry WS (1993). Waf1, a potential mediator of p53 tumor suppression. Cell.

[4] Polyak K (1994). p27Kip1, a cyclin-Cdk inhibitor, links transforming growth factor-beta and contact inhibition to cell cycle arrest. Genes & development.

[5] Toyoshima H, Hunter T (1994). p27, a novel inhibitor of G1 cyclin-Cdk protein kinase activity, is related to p21. Cell.

[6] Matsuoka S (1996). Imprinting of the gene encoding a human cyclin-dependent kinase inhibitor, p57KIP2, on chromosome 11p15. Proceedings of the National Academy of Sciences.

[7] Sherr CJ, Roberts JM (1999). CDK inhibitors: positive and negative regulators of G1-phase progression. Genes & development.

[8] Chen IT (1996). Characterization of p21Cip1/Waf1 peptide domains required for cyclin E/Cdk2 and PCNA interaction. Oncogene.

[9] Warbrick E (1998). PCNA binding through a conserved motif. Bioessays.

[10] Itoh Y, Masuyama N, Nakayama K, Nakayama KI, Gotoh Y (2007). The cyclin-dependent kinase inhibitors p57 and p27 regulate neuronal migration in the developing mouse neocortex. Journal of Biological Chemistry.

[11] Chang TS (2003). p57KIP2 modulates stress-activated signaling by inhibiting c-Jun NH2-terminal kinase/stress-activated protein Kinase. Journal of Biological Chemistry.

[12] Adkins JN, Lumb KJ (2002). Intrinsic structural disorder and sequence features of the cell cycle inhibitor p57Kip2. Proteins: Structure, Function, and Bioinformatics.

[13] Esteve V (2003). The structural plasticity of the C terminus of p21Cip1 is a determinant for target protein recognition. Chembiochem.

[14] Lacy ER (2004). p27 binds cyclin–CDK complexes through a sequential mechanism involving binding-induced protein folding. Nature Structural and Molecular Biology.

[15] Russo AA, Jeffrey PD, Patten AK, Massagué J, Pavletich NP (1996). Crystal structure of the p27Kip1 cyclin-dependent-kinase inibitor bound to the cyclin A–Cdk2 complex. Nature.

[16] Kriwacki RW, Hengst L, Tennant L, Reed SI, Wright PE (1996). Structural studies of p21Waf1/Cip1/Sdi1 in the free and Cdk2-bound state: conformational disorder mediates binding diversity. Proceedings of the National Academy of Sciences.

[17] Sivakolundu SG, Bashford D, Kriwacki RW (2005). Disordered p27Kip1 exhibits intrinsic structure resembling the Cdk2/cyclin A-bound conformation. Journal of molecular biology.

[18] Gulbis JM, Kelman Z, Hurwitz J, O’Donnell M, Kuriyan J (1996). Structure of the C-terminal region of p21WAF1/CIP1 complexed with human PCNA. Cell.

[19] Brown CJ (2002). Evolutionary rate heterogeneity in proteins with long disordered regions. Journal of molecular evolution.

[20] Xue B, Brown CJ, Dunker AK, Uversky VN (2013). Intrinsically disordered regions of p53 family are highly diversified in evolution. Biochimica et BiophysicaActa (BBA)-Proteins and Proteomics.

[21] Uversky V, Gillespie J, Fink A (2000). Why are “natively unfolded” proteins unstructured under physiologic conditions?. Proteins: Structure, Function, and Genetics.

[22] Romero P (2001). Sequence complexity of disordered protein. Proteins: Structure, Function, and Bioinformatics.

[23] Dunker A (2008). The unfoldomics decade: an update on intrinsically disordered proteins. BMC Genomics.

[24] Dunker AK, Brown CJ, Lawson JD, Iakoucheva LM, Obradović Z (2002). Intrinsic disorder and protein function. Biochemistry.

[25] Kovacs D, Tompa P (2012). Diverse functional manifestations of intrinsic structural disorder in molecular chaperones. Biochem. Soc. Trans..

[26] Van Der Lee R (2014). Classification of intrinsically disordered regions and proteins. Chemical reviews.

[27] Tompa P, Fuxreiter M (2008). Fuzzy complexes: polymorphism and structural disorder in protein–protein interactions. Trends in biochemical sciences.

[28] Tompa P, Schad E, Tantos A, Kalmar L (2015). Intrinsically disordered proteins: emerging interaction specialists. Current opinion in structural biology.

[29] Brown CJ, Johnson AK, Dunker AK, Daughdrill GW (2011). Evolution and disorder. Current opinion in structural biology.

[30] Gsponer J, Futschik ME, Teichmann SA, Babu MM (2008). Tight regulation of unstructured proteins: from transcript synthesis to protein degradation. Science.

[31] Malumbres M, Barbacid M (2005). Mammalian cyclin-dependent kinases. Trends in Biochemical Sciences.

[32] Nakayama KI, Nakayama K (1998). Cip/Kip cyclin‐dependent kinase inhibitors: brakes of the cell cycle engine during development. Bioessays.

[33] Watanabe H (1998). Suppression of cell transformation by the cyclin-dependent kinase inhibitor p57KIP2 requires binding to proliferating cell nuclear antigen. Proceedings of the National Academy of Sciences.

[34] Strzalka W, Ziemienowicz A (2010). Proliferating cell nuclear antigen (PCNA): a key factor in DNA replication and cell cycle regulation. Annals of botany.

[35] Morishita D, Katayama R, Sekimizu K, Tsuruo T, Fujita N (2008). Pim kinases promote cell cycle progression by phosphorylating and down-regulating p27Kip1 at the transcriptional and posttranscriptional levels. Cancer research.

[36] Podust VN (2000). A Nedd8 conjugation pathway is essential for proteolytic targeting of p27Kip1 by ubiquitination. Proceedings of the National Academy of Sciences.

[37] Kamura T (2003). Degradation of p57Kip2 mediated by SCFSkp2-dependent ubiquitylation. Proceedings of the National Academy of Sciences.

[38] Xie Q (1998). The sequence attribute method for determining relationships between sequence and protein disorder. genome Informatics.

[39] Williams RM (2000). The protein non-folding problem: amino acid determinants of intrinsic order and disorder. In Biocomputing.

[40] Hsu WL (2013). Exploring the binding diversity of intrinsically disordered proteins involved in one‐to‐many binding. Protein Science.

[41] Blenis J, Resh MD (1993). Subcellular localization specified by protein acylation and phosphorylation. Current opinion in cell biology.

[42] Liang J, Slingerland JM (2003). Multiple roles of the PI3K/PKB (Akt) pathway in cell cycle progression. Cell cycle.

[43] Child ES, Mann DJ (2006). The intricacies of p21 phosphorylation: protein/protein interactions, subcellular localization and stability. Cell cycle.

[44] Rodríguez-Vilarrupla A (2005). Binding of calmodulin to the carboxy-terminal region of p21 induces nuclear accumulation via inhibition of protein kinase C-mediated phosphorylation of Ser153. Molecular and cellular biology.

[45] Esteve-Puig R (2014). A mouse model uncovers LKB1 as an UVB-induced DNA damage sensor mediating CDKN1A (p21WAF1/CIP1) degradation. PLoS genetics.

[46] Zhao R (2013). CDK inhibitor p57Kip2 is downregulated by Akt during HER2-mediated tumorigenicity. Cell Cycle.

[47] Altschul SF, Gish W, Miller W, Myers EW, Lipman DJ (1990). Basic local alignment search tool. Journal of molecular biology.

[48] Pruitt KD, Tatusova T, Maglott DR (2005). NCBI Reference Sequence (RefSeq): a curated non-redundant sequence database of genomes, transcripts and proteins. Nucleic acids research.

[49] Katoh K, Misawa K, Kuma KI, Miyata T (2002). MAFFT: a novel method for rapid multiple sequence alignment based on fast Fourier transform. Nucleic acids research.

[50] Kumar S, Stecher G, Tamura K (2016). MEGA7: molecular evolutionary genetics analysis version 7.0 for bigger datasets. Molecular biology and evolution.

[51] Ronquist F (2012). MrBayes 3.2: efficient Bayesian phylogenetic inference and model choice across a large model space. Systematic biology.

[52] Miller, M., Pfeiffer, W. & Schwartz, T. Creating the CIPRES Science Gateway for inference of large phylogenetic trees. *Gatew. Comput. Environ. Work* 1–8 (2010).

[53] Jones DT, Taylor WR, Thornton JM (1992). The rapid generation of mutation data matrices from protein sequences. Bioinformatics.

[54] Tanabe AS (2011). Kakusan4 and Aminosan: two programs for comparing nonpartitioned, proportional and separate models for combined molecular phylogenetic analyses of multilocus sequence data. Molecular Ecology Resources.

[55] Dosztányi Z, Csizmok V, Tompa P, Simon I (2005). IUPred: web server for the prediction of intrinsically unstructured regions of proteins based on estimated energy content. Bioinformatics.

[56] Fuxreiter M, Tompa P, Simon I (2007). Local structural disorder imparts plasticity on linear motifs. Bioinformatics.

[57] Letunic I, Bork P (2016). Interactive tree of life (iTOL)v3: an online tool for the display and annotation of phylogenetic and other trees. Nucleic acids research.

[58] Blom N, Gammeltoft S, Brunak S (1999). Sequence and structure-based prediction of eukaryotic protein phosphorylation sites1. Journal of molecular biology.

[59] Drozdetskiy A, Cole C, Procter J, Barton GJ (2015). JPred4: a protein secondary structure prediction server. Nucleic acids research.

[60] Pupko T, Bell RE, Mayrose I, Glaser F, Ben-Tal N (2002). Rate4Site: an algorithmic tool for the identification of functional regions in proteins by surface mapping of evolutionary determinants within their homologues. Bioinformatics.

[61] Dos Santos HG, Nunez-Castilla J, Siltberg-Liberles J (2016). Functional diversification after gene duplication: paralog specific regions of structural disorder and phosphorylation inp53, p63, and p73. PloS one.

[62] Cohen O, Ashkenazy H, Belinky F, Huchon D, Pupko T (2010). GLOOME: gain loss mapping engine. Bioinformatics.

[63] Hao B (2005). Structural basis of the Cks1-dependent recognition of p27Kip1 by the SCFSkp2 ubiquitin ligase. Molecular cell.

[64] Kroker AJ, Bruning JB (2015). p21 exploits residue Tyr151 as a tether for high-affinity PCNA binding. Biochemistry.

