# Supplementary material for: Evolutionary Approach of Intrinsically Disordered CIP/KIP Proteins
Source: Sci Rep. 2019 Feb 7;9:1575. doi: 10.1038/s41598-018-37917-5 (PMC6367352; doi:10.1038/s41598-018-37917-5)
Supplement: Supplementary file 1 — Supplementary figure 1, Figure 2, Table 1, Table 2, and Table 3 [file 41598_2018_37917_MOESM1_ESM.docx]

EVOLUTIONARY APPROACH OF INTRINSICALLY DISORDERED CIP/KIP PROTEINS

Muhamad Fahmi^1^and *Masahiro Ito^1,2^

^1^Advanced Life Sciences Program, Graduate School of Life Sciences, Ritsumeikan University, 1-1-1 Nojihigashi, Kusatsu, Shiga 525-8577, Japan

^2^Department of Bioinformatics, College of Life Sciences, Ritsumeikan University, 1-1-1 Nojihigashi, Kusatsu, Shiga 525-8577, Japan

*maito@ed.ritsumei.ac.jp

**Supplementary Figures 1.** Boxplots of distribution of disorder propensity score. a, CDI domain; b, whole region.

**
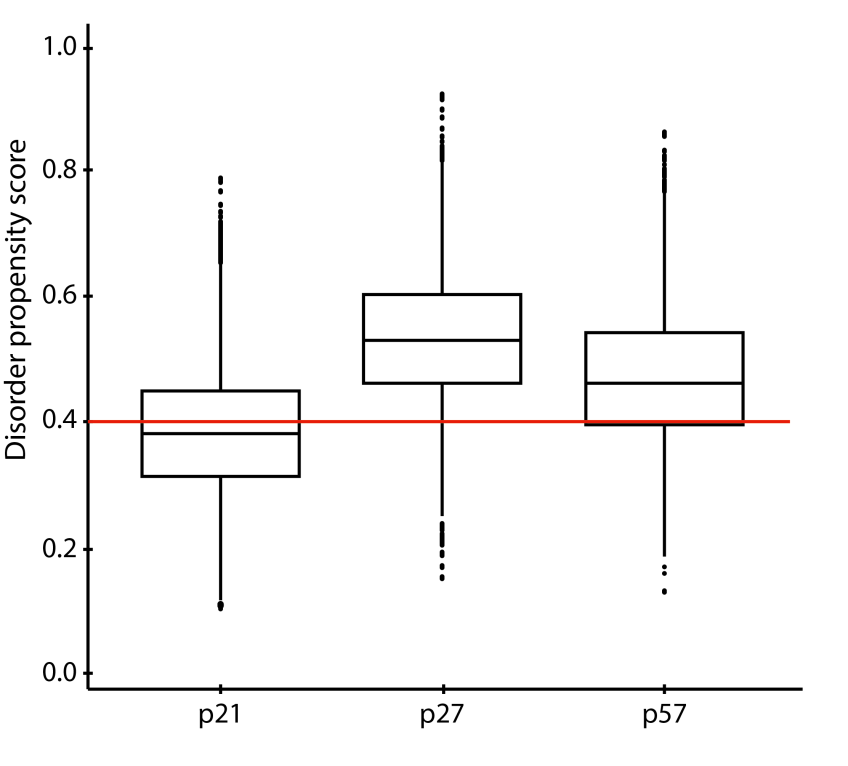
**

**a**

**
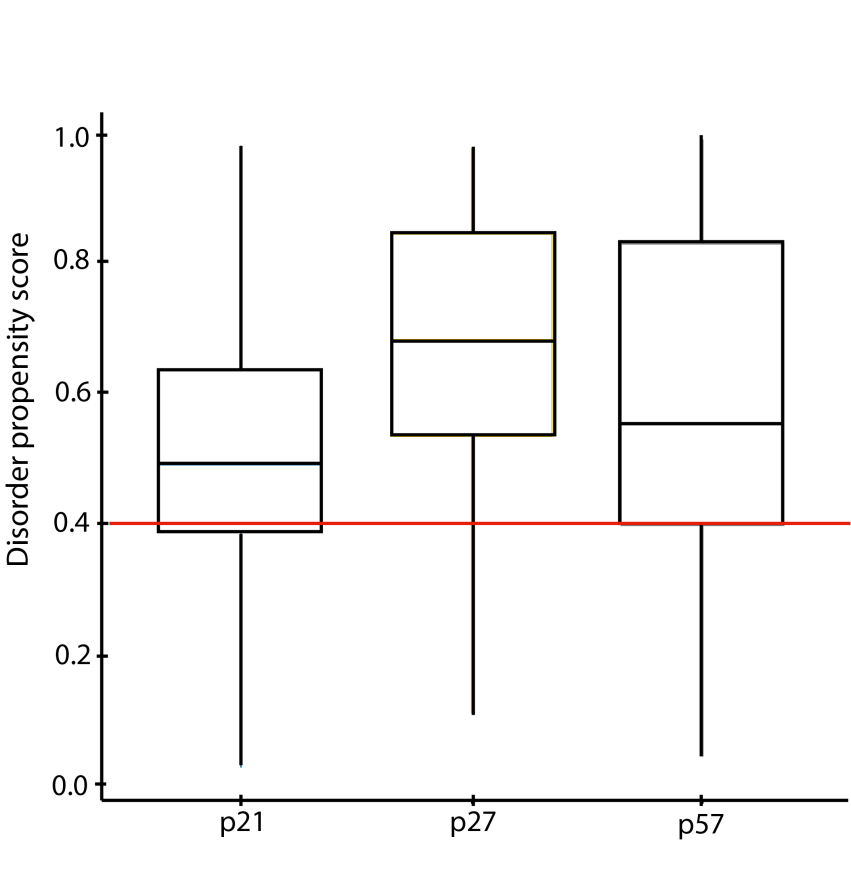
**

**b**

**Figure S2.** Heat-maps of the prediction of phosphorylation sites. The heat maps were plotted following the position of the aligned sites (columns) and the taxa position in the phylogenetic tree (rows). Colored boxes between the trees and heat maps indicate the taxonomic group according to the color guide in Fig.1. The heat maps show three colors, the white indicates non phosphorylated site, the red indicates phosphorylated site, and black indicates the gap. There is a domain bar above the heat map of each protein. In the domain bar, the green area indicates the CDI domain, and blue area indicates either PCNA or QT domain, whereas the black line indicates no domain. Three heat maps are shown in the figure: p21 (a), p27 (b), and p57 (c).

**
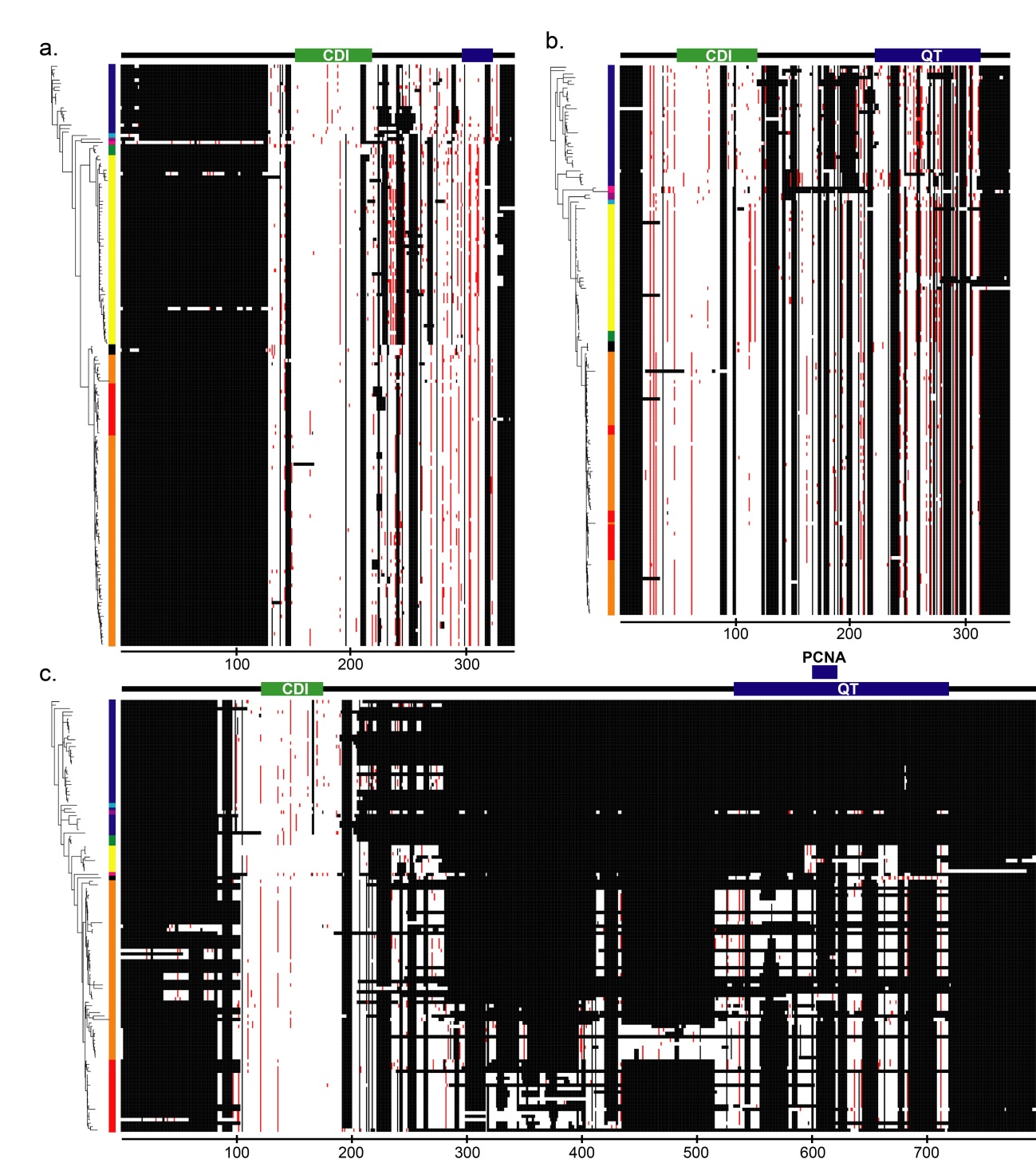
**

**Table S1.** p-distance of p21 representative dataset.

| **No** | **Species** | **1** | **2** | **3** | **4** | **5** | **6** | **7** | **8** | **9** | **10** |
| --- | --- | --- | --- | --- | --- | --- | --- | --- | --- | --- | --- |
| 1 | *Homo sapiens* |  |  |  |  |  |  |  |  |  |  |
| 2 | *Rhinopithecus roxellana* | 0.009 |  |  |  |  |  |  |  |  |  |
| 3 | *Mus musculus* | 0.159 | 0.159 |  |  |  |  |  |  |  |  |
| 4 | *Sarcophilus harrisii* | 0.29 | 0.299 | 0.355 |  |  |  |  |  |  |  |
| 5 | *Chrysemys picta bellii* | 0.495 | 0.495 | 0.486 | 0.449 |  |  |  |  |  |  |
| 6 | *Gallus gallus* | 0.523 | 0.523 | 0.514 | 0.505 | 0.374 |  |  |  |  |  |
| 7 | *Danio rerio* | 0.561 | 0.551 | 0.561 | 0.589 | 0.57 | 0.598 |  |  |  |  |
| 8 | *Xenopus laevis* | 0.579 | 0.579 | 0.57 | 0.57 | 0.57 | 0.617 | 0.589 |  |  |  |
| 9 | *Latimeria chalumnae* | 0.589 | 0.589 | 0.617 | 0.533 | 0.57 | 0.598 | 0.589 | 0.551 |  |  |
| 10 | *Callorhinchus milii* | 0.598 | 0.598 | 0.598 | 0.607 | 0.626 | 0.636 | 0.617 | 0.645 | 0.607 |  |

**Table S2.** p-distance of p27 representative dataset.

| **No** | **Species** | **1** | **2** | **3** | **4** | **5** | **6** | **7** | **8** | **9** | **10** |
| --- | --- | --- | --- | --- | --- | --- | --- | --- | --- | --- | --- |
| 1 | *Homo sapiens* |  |  |  |  |  |  |  |  |  |  |
| 2 | *Rhinopithecus roxellana* | 0.007 |  |  |  |  |  |  |  |  |  |
| 3 | *Mus musculus* | 0.072 | 0.08 |  |  |  |  |  |  |  |  |
| 4 | *Sarcophilus harrisii* | 0.21 | 0.217 | 0.239 |  |  |  |  |  |  |  |
| 5 | *Chrysemys picta bellii* | 0.21 | 0.217 | 0.239 | 0.225 |  |  |  |  |  |  |
| 6 | *Gallus gallus* | 0.217 | 0.225 | 0.225 | 0.239 | 0.109 |  |  |  |  |  |
| 7 | *Latimeria chalumnae* | 0.341 | 0.341 | 0.355 | 0.362 | 0.275 | 0.275 |  |  |  |  |
| 8 | *Callorhinchus milii* | 0.457 | 0.464 | 0.464 | 0.486 | 0.406 | 0.377 | 0.42 |  |  |  |
| 9 | *Danio rerio* | 0.478 | 0.478 | 0.464 | 0.507 | 0.5 | 0.471 | 0.471 | 0.507 |  |  |
| 10 | *Xenopus laevis* | 0.543 | 0.536 | 0.565 | 0.572 | 0.58 | 0.565 | 0.558 | 0.616 | 0.587 |  |

**Table S3.** p-distance of p57 representative dataset.

| No | Species | 1 | 2 | 3 | 4 | 5 | 6 | 7 | 8 | 9 | 10 |
| --- | --- | --- | --- | --- | --- | --- | --- | --- | --- | --- | --- |
| 1 | *Homo sapiens* |  |  |  |  |  |  |  |  |  |  |
| 2 | *Rhinopithecus roxellana* | 0 |  |  |  |  |  |  |  |  |  |
| 3 | *Mus musculus* | 0.181 | 0.181 |  |  |  |  |  |  |  |  |
| 4 | *Chrysemys picta bellii* | 0.319 | 0.319 | 0.347 |  |  |  |  |  |  |  |
| 5 | *Phascolarctos cinereus* | 0.333 | 0.333 | 0.319 | 0.278 |  |  |  |  |  |  |
| 6 | *Gallus gallus* | 0.389 | 0.389 | 0.347 | 0.236 | 0.347 |  |  |  |  |  |
| 7 | *Latimeria chalumnae* | 0.444 | 0.444 | 0.472 | 0.292 | 0.444 | 0.458 |  |  |  |  |
| 8 | *Nanorana parkeri* | 0.458 | 0.458 | 0.472 | 0.444 | 0.486 | 0.431 | 0.528 |  |  |  |
| 9 | *Rhincodon typus* | 0.472 | 0.472 | 0.472 | 0.25 | 0.431 | 0.361 | 0.333 | 0.472 |  |  |
| 10 | *Danio rerio* | 0.514 | 0.514 | 0.542 | 0.389 | 0.5 | 0.514 | 0.417 | 0.556 | 0.375 |  |
